# Nutrient intake differs among persons with celiac disease and gluten-related disorders in the United States

**DOI:** 10.1038/s41598-022-09346-y

**Published:** 2022-04-02

**Authors:** Aynur Unalp-Arida, Rui Liu, Constance E. Ruhl

**Affiliations:** 1 National Institute of Diabetes and Digestive and Kidney Diseases, Democracy 2, Room 6009, 6707 Democracy Boulevard, Bethesda, MD 20892-5458 USA; 2 Social and Scientific Systems, Inc, Silver Spring, MD USA; 3 Social and Scientific Systems, Inc., A DLH Holdings Corp Company, 8757 Georgia Ave., Silver Spring, MD 20910 USA; 4 Present Address: Sacred Heart University, 5151 Park Avenue, Fairfield, CT 06825 USA

**Keywords:** Gastroenterology, Gastrointestinal diseases

## Abstract

Persons with celiac disease (CD) may develop nutritional deficiencies, while individuals following a gluten-free diet (GFD) may lack essential nutrients. We examined nutrient intake from diet and supplements among persons with CD and GFD in the cross-sectional National Health and Nutrition Examination Survey, 2009–2014. Among 15,610 participants 20 years and older, we identified CD based on positive serology for immunoglobulin A against tissue transglutaminase, health care provider diagnosis, and adherence to a GFD. People without CD avoiding gluten (PWAG) adhered to a GFD without a diagnosis of CD. Two 24-h recalls assessed nutrient intake from diet and supplements. Compared to participants without CD or PWAG, persons with diagnosed CD had lower intake of total energy, carbohydrates, fat, and saturated and monounsaturated fatty acids. In contrast, persons with undiagnosed CD and positive serology had higher intake of those nutrients, sugar, and protein. Total carbohydrate and sugar intake was lower among PWAG. Persons with diagnosed CD had higher vitamin A and E intake, while those with undiagnosed CD had increased intake of calcium, phosphorus, magnesium, iron, zinc, copper, sodium, potassium, vitamin A, alpha-carotene, folic acid, and choline. Higher micronutrient intake with undiagnosed CD was observed more at high latitudes. PWAG had higher beta-carotene and lutein/zeaxanthin and lower folic acid intake. In the U.S. population over a 6-year period, total energy and macronutrient intake was decreased among persons with diagnosed CD, while intake of total energy, macronutrients, and multiple micronutrients was increased among persons with undiagnosed CD. Nutriomics studies of multiple analytes measured simultaneously across affected persons and populations are needed to inform screening for malabsorption and treatment strategies.

## Introduction

Celiac disease (CD) is a chronic immune-mediated disease of the small intestine triggered by dietary gluten in genetically predisposed individuals^1^. In an earlier report using National Health and Nutrition Examination Survey (NHANES) 2009–2014 data, CD prevalence in the U.S. was estimated as 0.7% overall and was higher among non-Hispanic whites (1.0%) than among other race-ethnicities (0.2%)^2^. The majority of celiac cases were undiagnosed in the NHANES population^3,4^. In addition, prevalence of people without CD avoiding gluten (PWAG) was 1.1% overall and did not differ by race-ethnicity^3^. Clinical presentation of CD and gluten-related disorders varies across individuals and by age^5^. Currently, the only CD treatment is lifelong strict adherence to a gluten-free diet (GFD)^6^. Previous studies suggest that because of the restrictive diet, celiac patients may experience imbalance in nutritional intake and consume less of certain macro- and micro-nutrients (fiber, thiamin, folate, calcium, magnesium), but more of others (fat, zinc, potassium, vitamin K)^5,7^. Therefore, assessment of changes in nutritional intake and deficiencies in celiac patients is critical in preventing complications from nutrient malabsorption and in improving quality of life^8^.

Reduced circulating blood concentrations of six micronutrients (iron, folate, vitamin B-12, vitamin D, zinc, magnesium) are common in untreated CD^9^. In a recent report, newly diagnosed CD patients in the U.S. were more likely to have serum deficiencies of zinc, albumin, copper, vitamin B-12, and folate compared with population controls^10^. Studies conducted in various countries assessing dietary habits of adult celiac patients reported reduced intake of certain vitamins and minerals, including folate^7,11^, potassium^7^, magnesium^7,12^, calcium^7^, zinc^7^, phosphate^7,11^, fiber^11^, niacin^11^, iodine, folic acid^12^, iron^12^, zinc^11^, and vitamins C^7^, D, B-1^12^, B-2^12^, B-6^12^, and B-12^11^, compared to age-matched study- and country-specific populations. Similar reduced micronutrient intake was reported in adult female celiac patients in Slovenia, compared to the general Central European population^5^, Although comparison groups in these studies were country-specific population controls, celiac patients were recruited using convenience sampling. Moreover, we are unaware of other studies of intake of a wide spectrum of dietary components in people with diagnosed and undiagnosed CD compared with PWAG and controls in a nationally representative population.

The NHANES 2009–2014 measured CD serology coupled with medical condition and GFD questions. Leveraging 6 years of serology data integrated with nutrient information from food and supplements, serum nutrient concentrations, and other health measures available in NHANES, we examined nutrient intake in participants with diagnosed and undiagnosed CD and PWAG compared with nationally representative adults without these conditions.

## Methods

### Study population

The NHANES is a series of nationally representative, cross-sectional surveys of the U.S. population with oversampling of non-Hispanic blacks, Hispanics, Asians (2011–2014), low income whites, and persons age 80 years or older that are conducted by the National Center for Health Statistics (NCHS) of the Centers for Disease Control and Prevention in 2-year cycles^13^. The survey includes in-home interviews, physical examinations, and laboratory data collected using a complex multistage, stratified, clustered probability sampling design. We analyzed survey data collected from 2009 through 2014, the years during which gluten-related disorders were assessed by interview and serology. All interviews and examinations were performed in accordance with relevant guidelines and regulations.

### Definitions of CD and gluten-avoidance without CD

NHANES 2009–2014 participants were asked the following interview questions: (1) Has a doctor or other health professional ever told you that you have celiac disease, also called sprue? and (2) Are you on a gluten-free diet?^14^ Serum specimens were shipped to the Celiac Disease Research Laboratory at Mayo Clinic, Rochester, MN, for serological testing. Serum was tested for tissue transglutaminase immunoglobulin A (tTG IgA) as a screening test (sensitivity, ~ 98%) with an enzyme-linked immunosorbent assay that uses human recombinant tTG (Inova Diagnostics, San Diego, CA, USA), and results were categorized as positive (> 10 U/ml), weakly positive (4–10 U/ml), or negative (< 4.0 U/ml)^4,15^.

**Figure 1:**
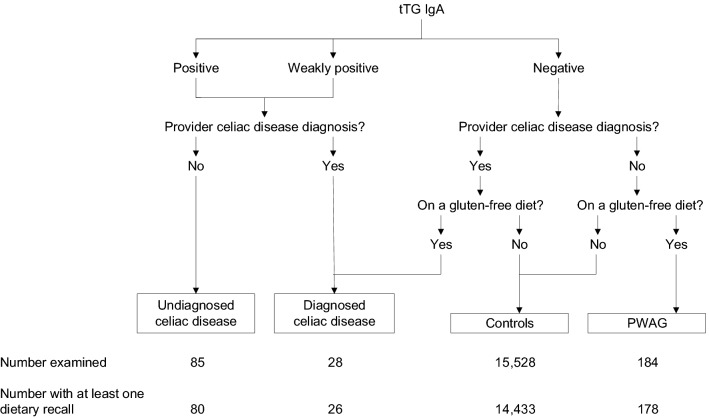
Case definitions and sample sizes for celiac disease, PWAG, and control groups for analyses of nutrient intake among persons with celiac disease and gluten-related disorders in NHANES, United States, 2009–2014. PWAG, persons without celiac disease avoiding gluten; tTG IgA, tissue transglutaminase immunoglobulin A.

Persons were identified as having diagnosed CD if they had a clinical (provider) diagnosis and either positive (or weakly positive) serology or adherence to GFD. Similarly, persons with undiagnosed CD had positive (or weakly positively) serology without a clinical diagnosis. PWAG was defined as adherence to GFD without a diagnosis of CD and with negative serology. Persons without CD and not on a GFD are referred to herein as ‘controls’. These case definitions are diagramed in Fig. 1 for reference. In contrast to previous reports, we included all NHANES 2009–2014 participants with positive tTG IgA alone, rather than serial test positivity to tTG IgA and endomysial antibody IgA, to increase the number of cases because of the low prevalence of CD in the general population^2,3^.

### Nutrient intake estimates

Dietary intakes were obtained from two 24-h dietary recall interviews in which respondents reported all foods and beverages consumed during the previous 24 h (midnight to midnight). The first dietary recall interview was collected in person during the Mobile Examination Center visit and the second interview was collected by telephone 3–10 days later. Detailed descriptions of the dietary interview methods and protocol are fully documented^16^. Daily nutrient and food energy intakes were estimated using the respective Food and Nutrient Database for Dietary Studies for participants in each NHANES cycle^17^.

### Supplement use

Information on dietary supplement use was collected by both a 30-day frequency questionnaire and two 24-h dietary recall interviews. For this analysis, we used supplement data from the 24-h recalls. Participants were queried about the 24-h period prior to the interview (midnight to midnight). Persons indicating using prescription or nonprescription dietary supplements as well as non-prescription antacids that contain calcium and magnesium were asked to show the interviewer the bottles of each supplement product taken. Quantity and duration of use were further queried during the interview. Daily nutrient intakes from dietary supplements and antacids were estimated using the NHANES Dietary Supplement Database. More information is provided in NHANES documentation^18^.

### Covariates

Data were collected on demographic and clinical characteristics and examined in relation to gluten-related disorders^19,20^: age (years), sex, race-ethnicity (non-Hispanic white, non-Hispanic black, Hispanic, other), education, income, and BMI. The highest grade of school completed was reported and categorized as less than high school graduation, a high school degree, or education beyond high school. Income was measured by the poverty income ratio (ratio of family income to poverty threshold) and categorized as tertiles (< 1.7, 1.7–<4.0, ≥ 4.0). Poverty income ratios of 1.7 and 4.0 represent family incomes of 1.7 times and 4 times, respectively, the poverty threshold for a given family size and year. BMI was categorized in kg/m^2^ as normal weight (< 25), overweight (25–< 30), or obese (≥ 30). Prevalence of CD and gluten-related disorders was previously found to vary by latitude in the United States; therefore, latitude was also included as a covariate to control for potential regional differences in nutrient intake^3^. Latitude (°North) was geocoded by NCHS from the participant’s residential address and categorized as < 35, 35–< 40, and ≥ 40 based on earlier findings^21^. Geographic data were used through the NCHS Research Data Center^22^. Serum was tested for hemoglobin, standard biochemistry, lipid profile, and selected vitamins and minerals as previously described^23^.

### Analytic sample

Of 25,426 NHANES 2009–2014 participants age 20 years and older, 16,966 (67%) attended a study visit at a mobile examination center and were included. A total of 15,610 (92%) participants further completed at least one of two 24-h dietary recall interviews and 13,825 participants (81% of those examined) provided two reliable dietary recalls. We included all individuals who completed at least one reliable 24-h dietary recall in analyses (Fig. 1). Persons with missing data on CD and PWAG status were excluded.

### Statistical analysis

Participant characteristics according to gluten-related disorders were compared using a chi-square (*χ*^2^) test and linear regression. Unadjusted means and standard errors of serum biochemical and nutritional markers were calculated according to CD and PWAG status using linear regression.

We estimated the distribution of usual nutrient intakes using methods developed by the National Cancer Institute (NCI)^24^. The NCI method is useful to estimate the within- and between-person variances and correct for the high within-person day-to-day variation commonly observed in 24-h recalls. A two-step process is involved in the NCI method. The first step uses the MIXTRAN macro to fit a nonlinear mixed effects model using the SAS NLMIXED procedure to obtain parameter estimates of mean usual intake. The data on amount were transformed to approximate normality using Box–Cox transformation. In the second step of the NCI method, the DISTRIB macro uses parameter estimates from MIXTRAN and a Monte Carlo method to estimate the distribution of usual intake of a nutrient^24^. To account for the complex survey sample design, variance estimation was carried out via the Balanced Repeated Replication technique with Fay’s modification^25^. Because intake of micronutrients can come from both diet and supplements, these two amounts were first summed for each participant recall day before applying the NCI method. Mean nutrient intakes were estimated unadjusted, adjusted for age, sex, and race-ethnicity, and additionally for education, income, and BMI. The NHANES-provided day one dietary recall weights were used in all nutrient intake analyses.

The relationship of nutrients with gluten-related disorders was further examined stratified by latitude using linear regression analysis (SUDAAN PROC REGRESS) to calculate (least squares) mean estimates adjusted for age, sex, and race-ethnicity. For these analyses, within person mean (2-day average) from the two 24-h food and supplement recalls was utilized because it was not feasible to use the NCI methods through the NCHS Research Data Center.

Multivariable-adjusted analyses excluded persons with missing values for any factor included in the model. *P*-values were two-sided, and a *P*-value of < 0.05 was considered to indicate statistical significance. All analyses utilized sample weights that accounted for unequal selection probabilities and nonresponse. Variance calculations for non-nutrient factors accounted for the design effects of the survey using Taylor series linearization^26^. SAS 9.4 (SAS Institute, Cary, NC, USA) and SUDAAN 11 (RTI, Research Triangle Park, NC, USA) were used for all analyses.

### Ethics approval and consent to participate

The NCHS Research Ethics Review Board approved the survey, and all participants provided written informed consent.

## Results

### Study population characteristics

Among 16,966 participants 20 years and older meeting eligibility criteria, 28 (0.2%) had diagnosed CD, 85 (0.5%) had undiagnosed CD, 184 (1.1%) were PWAG, and 15,528 (91.5%) did not have either condition and comprised the control group. We were unable to categorize 1141 (7%) participants with missing serology data. Sociodemographic characteristics by case status are summarized in Table 1. Compared to persons without CD and GFD (control group), those with diagnosed CD were older and more likely to be female and non-Hispanic white, while those with undiagnosed CD were younger, more likely to be non-Hispanic white, and did not differ by sex suggesting a differential health care seeking behavior in women or more symptomatic disease. PWAG were more likely to be female and did not differ by age or race-ethnicity. Persons with CD or PWAG had more education and income, and were less likely to be overweight.

**Table 1:** Characteristics of NHANES participants 20 years and older with and without celiac disease and PWAG, United States, 2009–2014.

Characteristic	Diagnosed celiac disease		Undiagnosed celiac disease		PWAG		Controls	
	N	%	N	%	N	%	N	%
**Age (years)**								
20–59	10	33.4	66	84.3	130	78.9	10,541	74.3
≥ 60	18	66.5*	19	15.7*	54	21.2	4987	25.8
**Sex**								
Male	6	18.2	43	53.2	80	36.0	7564	48.5
Female	22	81.8*	42	46.8	104	64.0*	7964	51.5
**Race-ethnicity**								
Non-Hispanic white	21	92.2	56	85.7	75	69.7	6758	67.3
Non-white	7	7.7*	29	14.3*	109	30.2	8770	32.7
**Education (years)**								
≤ 12	10	19.9*	27	24.4*	58	24.9*	7276	38.6
> 12	18	80.2	58	75.6	125	75.1	8235	61.4
**Income**^**a**^								
PIR 0–< 1.7	7	24.5	32	26.6	57	20.1*	6255	31.4
PIR 1.7–4.0	8	20.9	24	30.1	52	32.0	4395	33.4
PIR ≥ 4.0	11	54.6*	25	43.2	60	47.9*	3564	35.2
**BMI (kg/m**^**2**^**)**								
< 25	15	64.7*	32	37.0	76	44.0*	4525	29.8
25–< 30	5	14.8*	29	40.8	45	26.5	5077	33.7
≥ 30	7	20.5	23	22.2*	58	29.5	5753	36.5

NHANES, National Health and Nutrition Examination Survey; PIR, poverty income ratio; PWAG, persons without celiac disease avoiding gluten.

^a^PIR is family income to poverty threshold ratio. Cut points were tertiles.

*p < 0.05 compared with controls.

### Estimated usual nutrient intake

Of the survey participants included, 15,610 (92%) completed one dietary recall and 13,825 (81%) completed two recalls. Table 2 shows age-, sex- and race-ethnicity-adjusted estimated usual mean intakes of total energy and macronutrients derived from food sources by CD and PWAG status. Compared to persons without CD and GFD (control group), those with diagnosed CD had lower mean intake of total energy, total carbohydrates, total fat and saturated and monounsaturated fatty acids. In contrast, persons with undiagnosed CD had a higher mean intake of total energy, carbohydrates, sugar, protein, total fat, and saturated and monounsaturated fatty acids. Despite significantly increased macronutrient intake, persons with undiagnosed CD were less likely to be obese compared with controls. Mean total carbohydrates and sugar were lower among PWAG compared with controls. The results were similar with additional adjustment for education, income, BMI, and latitude (data not shown).

**Table 2:** Adjusted^a^ mean (SE) macronutrient intake from food among NHANES participants 20 years and older with and without celiac disease and PWAG, United States, 2009–2014.

Macronutrient	Diagnosed celiac disease (N = 26)	Undiagnosed celiac disease (N = 80)	PWAG (N = 178)	Controls (N = 14,433)
Total energy (kcal)	1773.5 (92.9)***	2594.0 (126.3)**	1995.5 (90.6)	2163.4 (11.3)
Total carbohydrates (g)	215.7 (11.0)***	305.9 (12.3)***	217.4 (16.5)*	260.5 (1.4)
Sugar	102.9 (7.7)	137.5 (5.3)***	91.7 (8.2)**	115.5 (0.9)
Fiber	17.9 (1.8)	19.8 (1.4)	20.9 (2.4)	17.5 (0.2)
Protein (g)	78.3 (6.0)	99.3 (5.2)**	82.7 (4.9)	83.6 (0.4)
Total fats (g)	68.5 (4.1)**	94.6 (5.0)*	81.9 (4.4)	81.9 (0.5)
Saturated fatty acids	20.6 (1.5)***	33.8 (2.3)**	24.6 (1.2)	26.5 (0.2)
Monounsaturated fatty acids	24.7 (1.9)*	33.5 (1.9)*	30.5 (2.0)	29.1 (0.2)
Polyunsaturated fatty acids	17.2 (1.4)	19.8 (1.0)	19.1 (1.5)	19.0 (0.1)
Cholesterol	275.0 (38.4)	339.4 (35.6)	267.9 (18.6)	284.6 (2.3)

NHANES, National Health and Nutrition Examination Survey; PWAG, persons without celiac disease avoiding gluten; SE, standard error.

^a^Adjusted for age, sex, and race/ethnicity.

*p < 0.05, **p < 0.01, and ***p < 0.001 compared to controls.

We further examined macronutrient intake among persons with diagnosed and undiagnosed CD and PWAG in comparisons by latitude (Table 3). Although the number of cases is small, for macronutrients adjusted for age, sex and race-ethnicity, the lower mean intake of total energy, total carbohydrates, total fat, and saturated and monounsaturated fatty acids observed among persons with diagnosed CD was found predominantly at a latitude of less than 35°N. In contrast, the higher mean intake of total energy, total carbohydrates, sugar, protein, total fat, and saturated and monounsaturated fatty acids found among persons with undiagnosed CD tended to be observed regardless of latitude, although most differences did not reach statistical significance due to smaller sample sizes. Similarly, among PWAG, lower mean total carbohydrate and sugar intake were seen regardless of latitude, although most differences were not statistically significant.

**Table 3:** Adjusted^a^ mean (SE) macronutrient intake from food among NHANES participants 20 years and older with and without celiac disease and PWAG by latitude, United States, 2009–2014.

Macronutrient latitude (ºN)	Diagnosed celiac disease (N = 26)	Undiagnosed celiac disease (N = 80)	PWAG (N = 178)	Controls (N = 14,433)
**Total energy (kcal)**				
< 35	1681.6 (59.3)*	2273.5 (267.2)	1921.5 (184.2)	2112.9 (16.2)
35– < 40	2164.5 (137.0)	2393.3 (131.0)*	1970.9 (121.4)	2089.1 (17.9)
≥ 40	1887.2 (116.3)	2375.5 (129.9)	2012.9 (121.5)	2117.7 (15.1)
**Total carbohydrates (g)**				
< 35	221.3 (12.8)*	298.8 (36.2)	235.6 (30.8)	256.1 (2.1)
35– < 40	267.1 (24.8)	285.9 (13.0)*	200.2 (21.8)*	253.1 (3.1)
≥ 40	217.7 (10.6)*	286.8 (17.2)	219.0 (18.2)	253.6 (1.9)
**Protein (g)**				
< 35	68.2 (8.9)	88.8 (11.3)	92.9 (20.0)	83.2 (0.6)
35– < 40	99.2 (7.7)*	88.5 (5.5)	94.3 (9.2)	81.8 (0.6)
≥ 40	86.4 (6.5)	96.5 (6.0)*	83.2 (6.2)	83.5 (0.6)
**Total fats (g)**				
< 35	60.6 (4.9)*	79.6 (8.9)	67.0 (6.1)*	79.8 (0.8)
35– < 40	81.4 (4.2)	88.5 (7.6)	86.4 (7.3)	79.5 (0.9)
≥ 40	69.1 (6.1)	85.5 (5.2)	85.6 (7.2)	79.8 (0.9)

NHANES, National Health and Nutrition Examination Survey; PWAG, persons without celiac disease avoiding gluten; SE, standard error.

^a^Adjusted for age, sex, and race/ethnicity.

*p < 0.05 compared with controls.

For micronutrients, unadjusted intake derived from food sources, and from food and supplements combined, is shown by disease status in Supplementary Table S1. The proportion of total intake from supplements varied by micronutrient. Table 4 shows age-, sex- and race-ethnicity-adjusted estimated usual intake of total micronutrients from food and supplements by CD and PWAG status. Persons with diagnosed CD had higher mean intake of vitamin A and vitamin E compared to persons without CD and GFD (control group). Persons with undiagnosed CD had higher mean intakes of calcium, phosphorus, magnesium, iron, zinc, copper, sodium, potassium, vitamin A, alpha-carotene, folic acid, folate and choline compared with controls. PWAG group had a higher mean intake of beta-carotene and lutein and zeaxanthin, and a lower mean intake of folic acid compared to persons without CD and GFD (control group). The results were similar with additional adjustment for education, income, BMI, and latitude (data not shown).

**Table 4:** Adjusted^a^ mean (SE) micronutrient intake from food and supplements among NHANES participants 20 years and older with and without celiac disease and PWAG, United States, 2009–2014.

Micronutrient	Diagnosed celiac disease (N = 26)	Undiagnosed celiac disease (N = 80)	PWAG (N = 178)	Controls (N = 14,433)
Calcium (mg)	1354.8 (138.0)	1421.7 (72.2)***	1171.0 (103.0)	1159.3 (7.9)
Phosphorus (mg)	1336.6 (90.8)	1694.5 (92.9)**	1414.4 (89.1)	1425.2 (7.1)
Magnesium (mg)	371.8 (22.0)	390.5 (17.9)**	419.8 (58.5)	336.7 (2.6)
Iron (mg)	20.2 (3.4)	23.5 (1.4)**	18.1 (1.5)	18.9 (0.2)
Zinc (mg)	15.8 (2.0)	18.8 (0.9)**	15.9 (1.7)	15.7 (0.1)
Copper (mg)	1.8 (0.2)	2.0 (0.1)**	1.8 (0.2)	1.6 (0)
Sodium (mg)	3043.2 (323.5)	4160.8 (201.6)**	3365.5 (196.5)	3583.5 (16.6)
Potassium (mg)	2865.6 (228.5)	3272.8 (119.8)***	3163.8 (311.0)	2766.4 (17.9)
Selenium (mcg)	118.2 (9.9)	148.9 (8.5)	119.5 (8.2)	132.6 (0.9)
Vitamin A (mcg)	808.3 (73.6)*	873.8 (71.9)**	781.0 (120.5)	652.3 (9.3)
Vitamin B-6 (mg)	5.4 (1.4)	4.8 (0.3)	5.4 (0.9)	4.4 (0.1)
Vitamin B-12 (mcg)	34.6 (16.9)	29.8 (4.3)	24.3 (7.6)	23.2 (1.1)
Vitamin C (mg)	194.5 (40.3)	159.5 (23.3)	261.6 (53.2)	157.8 (3.7)
Vitamin D (mcg)^b^	22.0 (3.9)	15.9 (1.5)	16.9 (3.1)	14.0 (0.3)
Vitamin E (mg)	10.9 (0.9)*	9.3 (0.7)	11.5 (1.3)	8.8 (0.1)
Vitamin K (mcg)	178.0 (36.9)	114.1 (9.2)	183.2 (40.1)	122.9 (2.2)
Beta-carotene (mcg)	3932.3 (822.0)	2716.8 (360.5)	4187.6 (781.5)*	2328.2 (59.0)
Alpha-carotene (mcg)	1227.1 (342.8)	851.0 (121.4)*	941.9 (356.7)	568.4 (19.2)
Beta-cryptoxanthin (mcg)	120.3 (19.7)	101.1 (14.6)	99.8 (14.4)	89.8 (2.2)
Lycopene (mcg)	5030.8 (1515.4)	8829.0 (1066.4)	6593.3 (780.9)	7096.9 (161.1)
Lutein + zeaxanthin (mcg)	2348.8 (670.6)	1650.8 (165.2)	3003.8 (596.3)*	1651.0 (52.3)
Thiamin (mg)	3.9 (0.9)	3.5 (0.2)	3.6 (0.5)	3.4 (0.1)
Riboflavin (mg)	4.9 (0.9)	4.2 (0.2)	4.2 (0.6)	3.9 (0.1)
Niacin (mg)	34.9 (5.5)	42.3 (3.6)	35.0 (4.0)	35.8 (0.4)
Folic acid (mcg)	292.7 (60.1)	411.2 (29.3)**	242.1 (20.3)***	323.4 (3.3)
Folate, DFE (mcg)	788.0 (80.8)	919.7 (59.7)*	756.3 (70.2)	776.6 (6.8)
Choline (mg)	346.2 (29.5)	406.5 (25.1)*	353.7 (22.1)	339.9 (2.1)

NHANES, National Health and Nutrition Examination Survey; PWAG, persons without celiac disease avoiding gluten; SE, standard error.

^a^Adjusted for age, sex, and race/ethnicity.

^b^Vitamin D2 + vitamin D3.

*p < 0.05, **p < 0.01, and ***p < 0.001 compared to controls.

As for macronutrients, we further examined total micronutrient intake among persons with diagnosed and undiagnosed CD and PWAG in comparisons by latitude (Table 5). For micronutrients adjusted for age, sex and race-ethnicity, the higher mean intake of calcium, phosphorus, magnesium, iron, zinc, copper, sodium, potassium, vitamin A, alpha-carotene, folic acid, folate and choline among participants with undiagnosed CD compared with controls tended to be observed regardless of latitude with the strongest relationships found at a latitude of greater than or equal to 40°N. The lower mean intake of folic acid among PWAG was also found primarily at the highest latitude.

**Table 5:** Adjusted^a^ mean (SE) total micronutrient intake from food and supplements among NHANES participants 20 years and older with and without celiac disease and PWAG by latitude, United States, 2009–2014.

Micronutrient latitude (ºN)	Diagnosed celiac disease (N = 26)	Undiagnosed celiac disease (N = 80)	PWAG (N = 178)	Controls (N = 14,433)
**Calcium (mg)**				
< 35	768.8 (214.6)	1427.2 (266.3)	1195.4 (193.4)	1063.8 (17.6)
35–< 40	1689.9 (274.4)*	1363.3 (175.8)	1091.8 (79.2)	1127.6 (14.9)
≥ 40	1345.2 (170.1)	1374.7 (64.4)*	1221.0 (147.0)	1216.6 (9.6)
**Phosphorus (mg)**				
< 35	1070.5 (16.8)*	1517.1 (199.1)	1515.8 (300.9)	1373.8 (11.1)
35–< 40	1583.2 (227.1)	1545.2 (87.4)	1486.5 (100.9)	1387.8 (9.7)
≥ 40	1434.7 (111.8)	1660.3 (78.0)*	1430.1 (110.8)	1441.8 (9.6)
**Magnesium (mg)**				
< 35	267.4 (72.7)	325.5 (58.5)	394.9 (60.4)	318.9 (4.6)
35–< 40	391.2 (61.5)	372.1 (28.0)	374.7 (27.1)	328.7 (6.1)
≥ 40	381.3 (18.2)*	376.4 (17.7)	536.8 (133.6)	342.1 (3.1)
**Iron (mg)**				
< 35	12.7 (7.4)	40.6 (15.4)	16.1 (1.3)	18.5 (0.3)
35–< 40	27.5 (6.5)	17.9 (2.2)	18.1 (2.6)	19.0 (0.4)
≥ 40	23.5 (4.5)	23.6 (1.6)*	20.1 (2.7)	19.4 (0.3)
**Zinc (mg)**				
< 35	4.9 (0.8)*	15.7 (2.7)	15.1 (3.1)	14.9 (0.2)
35–< 40	15.4 (4.1)	16.8 (2.2)	17.6 (2.0)	15.8 (0.2)
≥ 40	18.1 (3.8)	18.1 (0.7)*	17.9 (2.8)	16.2 (0.2)
**Copper (mg)**				
< 35	0.8 (0.2)*	1.5 (0.3)	1.6 (0.2)	1.5 (0)
35–< 40	2.2 (0.8)	1.7 (0.2)	1.9 (0.2)	1.6 (0)
≥ 40	1.8 (0.2)	3.0 (1.0)	2.0 (0.2)	1.6 (0)
**Sodium (mg)**				
< 35	2699.6 (83.2)*	3709.1 (346.4)	3100.7 (376.4)	3510.4 (30.5)
35–< 40	4329.4 (314.8)*	3578.6 (214.1)	3590.7 (250.2)	3524.3 (28.9)
≥ 40	3219.0 (266.8)	3989.5 (242.7)	3493.9 (305.0)	3534.2 (21.8)
**Potassium (mg)**				
< 35	2146.7 (463.5)	2764.9 (408.3)	2782.5 (245.5)	2629.1 (24.3)
35–< 40	3656.7 (358.4)*	3159.9 (226.8)*	3039.9 (148.4)*	2687.8 (30.1)
≥ 40	2854.2 (201.5)	3102.5 (88.5)*	3679.9 (611.5)	2833.3 (24.6)
**Selenium (mcg)**				
< 35	77.9 (10.8)*	125.9 (20.3)	134.4 (22.1)	133.5 (5.0)
35–< 40	176.9 (53.3)	125.4 (9.3)	133.8 (11.9)	137.5 (6.1)
≥ 40	119.3 (10.4)	147.5 (11.7)	118.8 (10.1)	132.9 (1.0)
**Vitamin A (mcg)**				
< 35	658.3 (34.9)	735.0 (219.6)	571.8 (98.1)	588.8 (17.5)
35–< 40	896.0 (131.5)	645.9 (42.1)	1012.1 (90.2)*	652.0 (12.9)
≥ 40	713.8 (91.0)	1527.7 (483.2)	1338.5 (596.7)	684.9 (9.3)
**Vitamin B-6 (mg)**				
< 35	5.8 (7.3)	7.0 (2.7)	13.0 (7.2)	6.4 (1.4)
35–< 40	5.4 (2.4)	3.0 (0.5)*	11.7 (3.7)	6.0 (0.4)
≥ 40	7.2 (5.1)	4.6 (1.4)	8.2 (2.8)	5.6 (0.3)
**Vitamin B-12 (mcg)**				
< 35	20.0 (58.7)	355.1 (262.6)	78.4 (53.1)	56.3 (8.8)
35–< 40	13.8 (10.1)*	25.2 (8.2)*	63.4 (23.3)	69.6 (7.6)
≥ 40	117.6 (132.3)	34.2 (10.0)	130.7 (74.5)	57.1 (7.0)
**Vitamin C (mg)**				
< 35	160.3 (140.7)	167.2 (64.8)	236.2 (43.8)	148.5 (6.1)
35–< 40	174.6 (24.2)	345.9 (201.4)	285.5 (67.1)	162.5 (7.0)
≥ 40	153.2 (31.2)	102.5 (20.4)*	639.6 (255.5)	178.3 (7.4)
**Vitamin D (mcg)**^**b**^				
< 35	28.0 (28.2)	13.6 (4.8)	35.3 (20.6)	11.7 (0.6)
35–< 40	19.8 (3.1)	15.8 (3.4)	28.2 (9.1)	15.9 (0.9)
≥ 40	12.4 (4.6)	12.9 (1.9)	20.8 (3.7)	17.2 (0.7)
**Vitamin E (mg)**				
< 35	7.1 (0.4)*	8.8 (1.6)	9.4 (1.1)	8.3 (0.2)
35–< 40	13.6 (1.6)*	8.2 (1.4)	9.5 (0.7)	8.8 (0.2)
≥ 40	10.4 (1.1)	9.1 (1.2)	14.2 (2.2)*	8.8 (0.1)
**Vitamin K (mcg)**				
< 35	118.6 (38.3)	98.2 (18.6)	124.8 (22.7)	116.0 (3.3)
35–< 40	319.1 (63.2)*	116.1 (9.8)	228.2 (48.1)*	124.8 (4.3)
≥ 40	164.5 (48.9)	87.0 (15.4)*	666.5 (486.9)	128.5 (4.5)
**Beta-carotene (mcg)**				
< 35	3907.3 (427.1)*	1794.4 (373.9)	2102.6 (538.0)	2019.4 (94.7)
35–< 40	2650.7 (1107.5)	2612.5 (290.2)	5655.3 (1273.2)*	2415.2 (116.7)
≥ 40	3349.8 (828.4)	2121.4 (477.0)	10,655.9 (6345.6)	2482.3 (69.6)
**Alpha-carotene (mcg)**				
< 35	1647.5 (139.4)*	377.0 (131.5)	403.6 (164.5)	384.6 (23.5)
35–< 40	684.5 (448.2)	516.7 (126.3)	723.1 (84.1)*	423.8 (20.7)
≥ 40	458.5 (211.8)	527.0 (144.6)	2358.7 (1838.0)	474.2 (19.8)
**Beta-cryptoxanthin (mcg)**				
< 35	71.2 (24.2)	143.9 (60.7)	97.8 (22.3)	80.1 (2.8)
35–< 40	89.7 (11.6)	126.3 (46.8)	95.7 (14.6)	87.6 (5.2)
≥ 40	68.5 (13.0)	69.9 (16.7)	116.9 (38.8)	90.4 (3.6)
**Lycopene (mcg)**				
< 35	8033.6 (6630.4)	6651.6 (1676.7)	4992.8 (1282.4)	4965.1 (141.3)
35–< 40	2585.9 (520.9)*	4282.7 (1348.2)	4796.2 (1605.8)	5030.2 (148.8)
≥ 40	3875.6 (847.8)*	5493.4 (1796.7)	7535.3 (1001.9)	5764.2 (219.6)
**Lutein + zeaxanthin (mcg)**				
< 35	586.8 (226.2)*	1211.7 (380.6)	1951.0 (404.8)	1606.1 (63.8)
35–< 40	2615.2 (634.5)	1543.0 (239.1)	3929.4 (1004.8)*	1907.5 (134.9)
≥ 40	2706.5 (1183.8)	1212.4 (214.8)*	7374.7 (4136.4)	1825.5 (71.2)
**Thiamin (mg)**				
< 35	5.6 (5.4)	1.4 (0.8)*	7.5 (4.0)	4.8 (0.4)
35–< 40	3.8 (1.2)	2.3 (0.3)*	10.1 (3.4)	5.2 (0.2)
≥ 40	5.8 (5.2)	3.1 (0.4)*	6.8 (2.2)	5.5 (0.4)
**Riboflavin (mg)**				
< 35	6.1 (6.0)	2.2 (0.6)*	7.7 (4.1)	4.0 (0.2)
35–< 40	4.8 (1.4)	2.5 (0.3)*	10.9 (3.5)	4.7 (0.2)
≥ 40	7.2 (5.0)	3.6 (0.2)*	5.7 (1.6)	5.0 (0.3)
**Niacin (mg)**				
< 35	40.9 (21.0)	30.9 (5.5)	31.3 (5.3)	34.0 (0.9)
35–< 40	44.6 (11.5)	31.8 (2.8)*	43.6 (6.7)	38.2 (0.9)
≥ 40	31.8 (6.7)	54.4 (16.2)	60.7 (28.1)	37.9 (0.9)
**Folic acid (mcg)**				
< 35	76.5 (19.5)*	701.2 (301.6)	325.3 (58.5)	297.3 (6.9)
35–< 40	457.9 (34.4)*	262.6 (72.2)	301.9 (66.4)	329.8 (7.8)
≥ 40	255.5 (71.3)	425.4 (25.6)*	255.0 (38.7)*	338.3 (5.6)
**Folate, DFE (mcg)**				
< 35	277.6 (80.6)*	1414.9 (524.5)	788.6 (114.1)	726.4 (13.8)
35–< 40	1149.2 (60.6)*	683.5 (115.8)	798.1 (123.8)	779.8 (15.3)
≥ 40	687.1 (106.5)	984.4 (55.9)*	811.2 (123.5)	805.4 (10.9)
**Choline (mg)**				
< 35	231.8 (14.9)*	343.3 (75.2)	406.7 (69.3)	336.8 (3.0)
35–< 40	460.8 (43.3)*	367.9 (30.3)	445.0 (45.5)*	328.6 (2.6)
≥ 40	363.5 (33.7)	411.0 (28.8)*	350.7 (30.6)	340.5 (3.7)

NHANES, National Health and Nutrition Examination Survey; PWAG, persons without celiac disease avoiding gluten; SE, standard error.

^a^Adjusted for age, sex and race/ethnicity.

^b^Vitamin D2 + vitamin D3.

*p < 0.05 compared with controls.

### Clinical laboratory and serum nutrition values

Lastly, we compared the clinical laboratory characteristics and serum concentrations of nutrition-related factors of participants with CD or PWAG to those of persons without these conditions (Table 6). Participants with diagnosed CD had lower unadjusted mean activities of alanine aminotransferase, aspartate aminotransferase, hemoglobin, creatine phosphokinase, gamma glutamyl transferase, lactate dehydrogenase, and selenium, and higher mean concentrations of HDL cholesterol. Among adults with undiagnosed CD, we observed lower mean gamma glutamyl transferase and folate concentrations. Compared with controls, PWAG had higher mean HDL cholesterol, potassium, and vitamin D concentrations.

**Table 6:** Unadjusted mean (SE) serum biochemical and nutritional markers among NHANES participants 20 years and older with and without celiac disease and PWAG, United States, 2009–2014.

	Diagnosed celiac disease		Undiagnosed celiac disease		PWAG		Controls	
	N	Mean (SE)	N	Mean (SE)	N	Mean (SE)	N	Mean (SE)
**Biochemistry profile and hemoglobin**								
Alanine aminotransferase (U/L)	26	20.5 (1.5)*	85	27.9 (2.0)	184	25.3 (2.6)	15,513	25.5 (0.2)
Albumin (g/dL)	26	4.2 (0.1)	85	4.3 (0.1)	184	4.3 (0.0)	15,518	4.3 (0.0)
Alkaline phosphatase (U/L)	26	68.8 (3.5)	85	68.8 (2.4)	184	68.4 (2.4)	15,515	66.2 (0.3)
Aspartate aminotransferase (U/L)	26	23.5 (1.0)*	85	28.0 (1.5)	184	24.7 (1.3)	15,509	25.8 (0.2)
Bicarbonate (mmol/L)	26	25.9 (0.5)	85	24.9 (0.4)	184	25.1 (0.3)	15,516	25.3 (0.1)
Blood urea nitrogen (mg/dL)	26	13.5 (0.8)	85	12.9 (0.6)	184	13.4 (0.6)	15,517	13.2 (0.1)
Hemoglobin (g/dL)	27	13.6 (0.2)*	85	14.0 (0.2)	184	14.0 (0.1)	15,501	14.2 (0.0)
Calcium (mg/dL)	26	9.5 (0.1)	85	9.3 (0.1)	184	9.5 (0.0)	15,487	9.4 (0.0)
Total cholesterol (mg/dL)	27	193.9 (6.9)	85	188.1 (5.5)	184	198.5 (4.5)	15,528	193.6 (0.5)
HDL cholesterol (mg/dL)	27	62.7 (4.0)*	85	50.6 (2.1)	184	58.2 (1.5)*	15,528	53.0 (0.2)
Triglyceride (non-fasting; mg/dL)	26	125.0 (17.0)	85	131.2 (12.6)	184	133.6 (10.7)	15,507	153.0 (1.7)
Creatinine (mg/dL)	26	0.9 (0.0)	85	0.8 (0.0)	184	1.0 (0.1)	15,518	0.9 (0.0)
Creatine phosphokinase (U/L)^a^	20	102.5 (10.8)*	49	178.2 (56.7)	140	123.4 (10.5)	9939	141.2 (2.3)
Gamma glutamyl transferase (U/L)	26	20.5 (2.7)*	85	21.4 (1.8)*	184	27.0 (4.2)	15,515	27.2 (0.4)
Glucose (non-fasting; mg/dL)	26	94.2 (4.3)	85	99.6 (6.3)	184	102.1 (3.3)	15,518	100.0 (0.4)
Iron (ug/dL)	26	90.1 (7.9)	85	85.1 (4.4)	184	90.5 (3.1)	15,491	86.5 (0.5)
Lactate dehydrogenase (U/L)	26	120.2 (3.4)*	85	122.3 (3.1)	184	124.1 (2.5)	15,509	127.6 (0.5)
Phosphorus (mg/dL)	26	3.9 (0.1)	85	3.9 (0.0)	184	3.8 (0.1)	15,513	3.8 (0.0)
Potassium (mmol/L)	26	4.1 (0.1)	85	4.1 (0.0)	184	4.1 (0.0)*	15,514	4.0 (0.0)
Chloride (mmol/L)	26	103.7 (0.6)	85	103.7 (0.5)	184	104.0 (0.3)	15,516	104.0 (0.1)
Sodium (mmol/L)	26	139.0 (0.6)	85	138.7 (0.5)	184	139.4 (0.2)	15,516	139.3 (0.1)
Total bilirubin (mg/dL)	26	0.7 (0.1)	85	0.7 (0.0)	184	0.7 (0.0)	15,505	0.7 (0.0)
Total protein (g/dL)	26	6.8 (0.1)	85	7.1 (0.1)	184	7.1 (0.1)	15,488	7.1 (0.0)
Uric acid (mg/dL)	26	5.0 (0.3)	85	5.4 (0.2)	184	5.4 (0.1)	15,514	5.4 (0.0)
**Vitamins and minerals**								
Vitamin B-6 (nmol/L)^b^ (pyridoxal 5’-phosphate)	6	126.0 (69.8)	36	66.1 (6.5)	43	93.2 (16.1)	5370	71.5 (1.9)
Vitamin B-12 (pg/mL)^a^	20	610.6 (62.7)	49	660.6 (203.1)	140	785.0 (116.6)	9946	615.1 (7.0)
Vitamin D (nmol/L)	27	79.7 (5.1)	85	73.1 (3.3)	184	84.9 (3.4)*	15,527	69.2 (0.8)
Folate (ng/mL)	27	18.9 (2.7)	85	16.9 (1.1)*	184	23.5 (3.5)	15,453	19.8 (0.2)
Ferritin (ng/mL)^c^	0	–	10	37.6 (13.1)	12	76.9 (20.2)	1537	59.6 (1.2)
Copper (µg/dL)^d^	8	116.5 (2.5)	14	128.0 (12.4)	39	115.0 (5.4)	3274	118.4 (1.1)
Selenium (µg/L)^d^	8	112.2 (5.0)*	14	121.3 (5.0)	39	127.5 (3.5)	3273	130.4 (0.8)
Zinc (µg/dL)^d^	8	75.1 (6.0)	14	78.0 (3.2)	39	80.6 (3.7)	3274	82.2 (0.6)

NHANES, National Health and Nutrition Examination Survey; PWAG, persons without celiac disease avoiding gluten; SE, standard error.

^a^Measured in NHANES 2011–2014.

^b^Measured in NHANES 2009–2010.

^c^Measured on females 12–49 years in NHANES 2009–2010.

^d^Measured from a one-third sample in NHANES 2011–2014.

*p < 0.05 compared to controls.

## Discussion

In the U.S. population, persons with undiagnosed CD had higher intake of multiple macro- and micronutrients, including total energy, carbohydrates, sugar, protein, fat, saturated and monounsaturated fatty acids, calcium, phosphorus, magnesium, iron, zinc, copper, sodium, potassium, vitamin A, alpha-carotene, folic acid, folate and choline with no evident increased obesity. Higher nutrient intake among persons with undiagnosed CD suggests compensation for potential malabsorption. In contrast, persons with diagnosed CD had lower intake of total energy, carbohydrates, fat, and saturated and monounsaturated fatty acids, and higher intake of vitamin A and vitamin E compared with controls. This could be due to imbalances in nutritional intake resulting from GFD adherence, though the small number of participants with diagnosed CD requires caution in interpreting results. Persons with diagnosed CD had lower BMI compared with controls. With healing of the intestinal mucosa on a GFD, nutrient absorption would be expected to increase and added calories could lead to weight gain. However, we did not have information on length of time on a GFD or diet adherence. There have been concerns about potential for weight gain on a GFD due to nutritional content, but that is not suggested by our findings^27,28^. PWAG had lower intake of carbohydrates, sugar, and folic acid, and higher intake of beta-carotene and lutein/zeaxanthin. Among available serum nutritional measures, persons with diagnosed CD had decreased hemoglobin and selenium concentrations, persons with undiagnosed CD had lower folate concentrations despite increased dietary intake, and PWAG had increased vitamin D concentrations compared with controls. Some serum markers were not measured on the entire NHANES sample, which could have limited ability to detect differences from controls (Table 6). We are unaware of other studies of a wide spectrum of macro- and micronutrients in people with diagnosed and undiagnosed CD and PWAG compared with controls in a nationally representative population.

Previously, we found a lower prevalence of CD and gluten-related disorders in persons living in southern compared with northern latitudes of the United States^3^. In the present analysis, we examined nutrient intake by latitude of residence. Lower intake of total energy and macronutrients with diagnosed CD was found predominantly at low latitudes. Higher micronutrient intake with undiagnosed CD was observed more at high latitudes. However, these results should be interpreted with caution because of small numbers of participants at lower latitudes particularly among persons with diagnosed CD.

Macro- and micronutrient deficiencies are frequently found in newly diagnosed and untreated CD, including deficiencies of iron, vitamin D, calcium, vitamin B-12, folate, zinc and vitamin B-6^29^. This could be due to malabsorption caused by underlying etiologies in affected persons and may lead to compensatory increase in macro- and micronutrient intake possibly accounting for higher intake of multiple nutrients among persons with undiagnosed CD in the current analysis. Risk of nutritional deficiency in CD is compounded by nutritional inadequacy of the traditional GFD in which removal of gluten-containing grains can diminish nutrient content, specifically of minerals, B vitamins, and fiber^29^. Because the most popular gluten-free raw materials are mineral poor, deficiencies of calcium, iron, magnesium, and zinc are among the most common^30^. Gluten-free processed foods have become more widely available, leading to increased concerns regarding nutritional quality of a GFD^31^.

The gluten-free market is growing faster than CD prevalence, indicating increasing use of the GFD in the absence of a CD diagnosis^29^. This should be a source of concern given the potential for nutritional imbalances with the GFD. In a recent report on non-celiac gluten sensitivity, 65 adults with self-reported sensitivity on GFD had a high proportion of energy from fat, while intakes of vitamin D, folic acid, calcium, iodine, and iron were lower than recommended^32^. PWAG may include persons with non-celiac gluten sensitivity, those with CD who instituted a GFD before diagnosis, and those without CD diagnosis who follow a GFD for symptomatic relief. We did not have information on reason for GFD, so were unable to distinguish between persons with non-celiac gluten sensitivity and those following a GFD for other reasons.

A limitation of 24-h dietary recall data is potential for systematic and random measurement error; however, analytic methodology used in this analysis assumes that the 24-h recall is an unbiased instrument. As previously reported, a limitation of using NHANES to study CD is inability to confirm the diagnosis by small intestinal histology^3^. However, upper endoscopy with small intestinal biopsy would be difficult to perform on the general U.S. population. We defined CD based on a self-reported health care provider diagnosis and adherence to a GFD or on positive serology. In contrast to previous reports, we included all participants with positive tTG IgA alone, rather than serial test positivity, to increase case number because of low prevalence of CD in the general population^2,3^. This resulted in 34 additional cases with undiagnosed CD, but may have decreased specificity. However, in a sensitivity analysis with CD defined using serial serology, nutrient relationships were similar to the main analysis. In addition, accuracy of clinical diagnosis among participants with negative serology is unknown. Serology becomes unreliable among persons who adopt a GFD prior to a formal celiac disease diagnosis^33^. Another limitation of the survey was the small number of cases with gluten-related disorders despite 6 years of NHANES testing. Furthermore, because of the large number of comparisons, some associations could be the result of chance; therefore, results should be interpreted with caution. However, NHANES is the first nationally representative survey to collect CD serology. The limitations are balanced by the benefits of a large, national, population-based sample, particularly avoidance of ascertainment bias found in clinical studies of selected patients and ability to generalize results to the U.S. population.

## Conclusions

Among U.S. adults, total energy and macronutrient intake tended to be lower with diagnosed CD. In contrast, persons with undiagnosed CD and positive serology had higher intake of total energy, macronutrients, and multiple micronutrients, including calcium, phosphorus, magnesium, iron, zinc, copper, sodium, potassium, vitamin A, alpha-carotene, folic acid, and choline. PWAG had lower folic acid intake. Nutriomics studies of multiple analytes measured simultaneously across affected persons and populations are needed to inform screening for malabsorption and treatment strategies.

## Supplementary Information

Supplementary Table S1.

## Data Availability

The NHANES datasets analyzed during the current study are publicly available from the National Center for Health Statistics (NCHS) (https://www.cdc.gov/nchs/nhanes/index.htm), except for geographic data (latitude) that are restricted to use through the NCHS Research Data Center (http://www.cdc.gov/rdc/) per NCHS, Centers for Disease Control and Prevention policy.

## Abbreviations

CD: Celiac disease
GFD: Gluten-free diet
NCI: National Cancer Institute
NCHS: National Center for Health Statistics
NHANES: National Health and Nutrition Examination Survey
PWAG: People without CD avoiding gluten
tTG IgA: Tissue transglutaminase immunoglobulin A
