# Supplementary material for: Nutrient intake differs among persons with celiac disease and gluten-related disorders in the United States
Source: Sci Rep. 2022 Apr 2;12:5566. doi: 10.1038/s41598-022-09346-y (PMC8976850; doi:10.1038/s41598-022-09346-y)
Supplement: Supplementary file 1 — Supplementary Table S1. [file 41598_2022_9346_MOESM1_ESM.pdf]

Nutrient intake differs among persons with celiac disease and gluten-related disorders in the United States

Aynur Unalp-Arida, M.D., Ph.D.[1]
Rui Liu, Ph.D.[2,3]
Constance E. Ruhl, M.D., Ph.D.[4,5]

[1]National Institute of Diabetes and Digestive and Kidney Diseases
Democracy 2, Room 6009
6707 Democracy Boulevard
Bethesda, MD 20892-5458
301-594-8879 phone
301-480-8300 fax
aynur.unalp-arida@nih.gov

[2]Affiliation where work was conducted:
Social & Scientific Systems, Inc,
Silver Spring, Maryland

[3]Present affiliation:
Sacred Heart University
5151 Park Avenue
Fairfield, CT 06825
liur3@sacredheart.edu

[4]Social & Scientific Systems, Inc., a DLH Holdings Corp company
8757 Georgia Ave.
Silver Spring, Maryland 20910
301-628-3272
cruhl@s-3.com
https://orcid.org/0000-0002-8160-9859

[5]Address for correspondence and reprint requests.

Supplementary Table 1. Unadjusted mean (SE) micronutrient intake from food and supplements among NHANES participants 20 years and older with and without celiac disease and PWAG, United States, 2009-2014

	Diagnosed celiac disease (N=26)		Undiagnosed celiac disease (N=80)		PWAG (N=178)		Controls (N=14,433)	
Micronutrient	Food Only	Food + Supplements	Food Only	Food + Supplements	Food Only	Food + Supplements	Food Only	Food + Supplements
Calcium (mg)	977.6 (82.7)	1383.2 (156.8)	1282.6 (61.4)***	1447.0 (72.7)***	977.5 (84.5)	1167.3 (114.4)	998.0 (6.2)	1159.1 (8.0)
Phosphorus (mg)	1333.2 (95.2)	1347.6 (95.2)	1723.1 (77.4)***	1729.0 (77.2)***	1398.2 (92.3)	1406.8 (92.8)	1418.3 (6.8)	1425.3 (6.9)
Magnesium (mg)	321.8 (16.2)	374.9 (22.1)	359.7 (18.0)**	392.3 (17.4)**	367.1 (30.9)	424.4 (55.9)	310.3 (2.2)	336.7 (2.6)
Iron (mg)	14.1 (2.4)	20.4 (3.6)	18.4 (1.0)**	23.5 (1.4)**	14.7 (1.2)	18.1 (1. 5)	15.3 (0.1)	18.9 (0.2)
Zinc (mg)	10.1 (0.7)*	15.9 (2.2)	14.6 (0.7)***	18.9 (1.0)**	11.8 (0.8)	16.0 (1.7)	11.6 (0.1)	15.7 (0.1)
Copper (mg)	1.4 (0.1)	1.8 (0.2)	1.6 (0.1)**	2.0 (0.1)**	1.6 (0.1)	1.8 (0.2)	1.3 (0.01)	1.6 (0)
Sodium (mg)	3063.4 (318.7)	3063.6 (319.0)	4127.3 (208.6)*	4127.5 (208.5)*	3363.3 (194.7)	3370.1 (194.9)	3582.6 (16.4)	3584.7 (16.5)
Potassium (mg)	2836.5 (247.0)	2862.2 (248.6)	3244.1 (107.2)***	3262. 9 (108.3)***	3141.3 (308.2)	3183.8 (312.3)	2752.4 (17.4)	2767.2 (17.5)
Selenium (mcg)	96.8 (7.9)*	117.1 (12.3)	132.4 (7.9)*	148.7 (9.1)	105.6 (5.2)	119.8 (8.0)	115.5 (0.7)	132.6 (0.9)
Vitamin A (mcg)†	828.9 (82.1)*	--	893.6 (69.2)**	--	794.5 (125.4)	--	652.0 (9.4)	--
Vitamin B-6 (mg)	2.3 (0.3)	5.5 (1.4)	2.5 (0.1)**	4.7 (0.3)	2.3 (0.2)	5.4 (0.9)	2.2 (0.2)	4.4 (0.1)
Vitamin B-12 (mcg)	5.5 (0.8)	35.3 (15.9)	7.5 (0.9)*	30.0 (4.4)	4.4 (0.3)*	24.7 (7.6)	5.2 (0.1)	23.3 (1.1)
Vitamin C (mg)	103.8 (24.3)	198.1 (45.9)	82.0 (8.9)	160.6 (24.4)	121.0 (17.3)	262.0 (52.5)*	84.5 (1.6)	157.8 (3.7)
Vitamin D (mcg)‡	6.2 (0.7)	22.6 (4.4)	5.8 (0.5)*	16.0 (1.5)	3.9 (0.3)**	17.0 (3.1)	4.8 (0.1)	14.0 (0.3)
Vitamin E (mg)†	11.0 (1.0)*	--	9.3 (0.7)	--	11.5 (1.3)	--	8.8 (0.1)	--
Vitamin K (mcg)	160.6 (38.0)	182.2 (40.1)	106.6 (8.6)	114.0 (9.3)	174.6 (38.1)	183.1 (40.1)	115.2 (2.0)	122.9 (2.2)
Beta-carotene (mcg)†	4013.4 (862.0)	--	2734.8 (378.3)	--	4238.1 (797.8)*	--	2326.4 (58.9)	--
Alpha-carotene (mcg)†	1236.7 (326.0)	--	843.2 (116.6)*	--	935.8 (341.5)	--	566.9 (19.3)	--
Beta-cryptoxanthin (mcg)†	121.1 (19.6)	--	101.1 (15.0)	--	100.1 (14.3)	--	89.8 (2.2)	--
Lycopene (mcg)	5004.8 (1646.1)	5745.4 (1907.8)	8861.0 (1177.9)	9835.8 (1364.7)	6607.9 (828.6)	6989.2 (800.6)	7128.3 (159.8)	7771.1 (205.5)
Lutein + zeaxanthin (mcg)	2235.9 (588.3)	2324.9 (619.5)	1595.3 (159.1)	1659.7 (162.2)	2798.1 (561.5)*	3007.9 (605.6)*	1536.6 (46.1)	1651.3 (52.5)
Thiamin (mg)	1.5 (0.2)	3.9 (0.8)	2.0 (0.1)***	3.5 (0.2)	1.4 (0.1)*	3.6 (0.5)	1.7 (0.01)	3.4 (0.1)
Riboflavin (mg)	2.4 (0.2)	5.0 (1.0)	2.7 (0.2)**	4.2 (0.2)	2.1 (0.1)	4.2 (0.6)	2.2 (0.01)	3.9 (0.1)
Niacin (mg)	23.2 (2.9)	35.1 (5.7)	31.3 (1.4)***	42.1 (3.7)	23.8 (1.1)*	35.2 (4.1)	26.3 (0.2)	35.8 (0.4)

Folic acid (mcg)	165.6 (51.3)	287.5 (69.5)	257.3 (20.7)**	415.5 (34.5)**	100.4 (11.5)***	235.6 (21.6)***	192.9 (2.2)	323.6 (3.3)
Folate, DFE (mcg)	565.7 (78.7)	782.8 (109.4)	681.1 (42.8)**	946.7 (66.1)*	510.6 (53.2)	749.0 (79.6)	553.6 (5.0)	776.7 (6.8)
Choline (mg)	351.3 (32.6)	352.4 (32.9)	411.4 (26.4)**	411.4 (26.4)*	350.6 (22.4)	354.7 (23.3)	338.5 (2.1)	339.9 (2.1)

NHANES, National Health and Nutrition Examination Survey; PWAG, persons without celiac disease avoiding gluten; SE, standard error.

†Intake from food, but not supplements was measured.

‡Vitamin D2 + vitamin D3.

*p<0.05, **p<0.01, and ***p<0.001 compared to controls.
